# Independent and combined effect of serum copper and folate on depression: cross-sectional data from the NHANES 2011–2016

**DOI:** 10.3389/fnut.2024.1389480

**Published:** 2024-08-09

**Authors:** Mengqing Liu, Gang Wang, Chongfei Jiang

**Affiliations:** ^1^College of Humanities and Management, Guilin Medical College, Guilin, China; ^2^Department of Nephrology, The University of HongKong-ShenZhen Hospital, ShenZhen, Guangdong, China

**Keywords:** NHANES, folate, copper, depression, interaction effect

## Abstract

**Background and objective:**

Depression is a widespread mental health condition that can impact both mental and physical well-being. Prior research has shown that high levels of copper in the blood and low levels of folate are linked to depression. This study aimed to explore whether serum folate levels, independently or in combination with serum copper levels, associated with the risk of depression.

**Methods:**

Data from participants aged 18–80 years in the National Health and Nutrition Examination Survey (NHANES) from 2011 to 2016 were analyzed to examine the role of trace elements. Depression was assessed using the nine-item Patient Health Questionnaire (PHQ-9). Logistic regression analyses were employed to evaluate the main effect of serum copper and folate levels on depression. Three indices, including the relative excess risk of interaction (RERI), attributable proportion of interaction (API), and synergy index (SI), were used to analyze the interaction effect.

**Results:**

Among the 4,847 participants selected for this study, 429 (8.9%) had PHQ-9 scores above 10, which we defined as having depressive symptoms. After adjusting for all confounding factors, higher serum copper levels [≥15.5 vs. <15.5 μmol/L, odds ratio (OR): 1.54; 95% confidence intervals (CI): 1.18–2.11] and folate deficiency (folate ≥53.7 vs. <53.7 μmol/L, OR: 1.44; 95% CI: 1.21–2.10) were associated with an increased risk of depression. Patients with both higher serum copper levels and folate deficiency (OR: 2.11; 95% CI: 1.43–3.14) had the highest risk of depression than other levels. High copper levels and low folate levels are associated with the occurrence of depression symptoms, and there may be a synergistic effect between them (SI: 1.65; 95% CI: 1.49–4.76), with this interaction accounting for 19% of depression cases (API: 0.19; 95% CI: 0.01–0.54).

**Conclusion:**

There may be a synergistic interaction between high copper levels and low folate levels associated with increasing risk of depression. Further population-based interventional studies are needed to confirm whether folic acid supplementation is effective in preventing depression in individuals with high blood copper levels.

## Introduction

Depression is a common chronic illness worldwide that can impair normal functioning, cause depressive thoughts, and negatively impact quality of life. Patients with major depressive disorder are at increased risk of cardiovascular disease and poor treatment outcomes, as well as increased morbidity and mortality (1–3). The number of new cases of depression worldwide rose from 172 million in 1990 to 258 million in 2017, representing an increase of 49.86% (4). The World Health Organization had predicted that by 2020, depression will rank second in disability-adjusted life years calculated for all ages. Despite a decrease in the age-standardized rate (ASR) of dysthymia in the United States, the country still had the highest ASR in 2017 (4). This indicates that the United States should pay more attention to this issue and continue to implement measures to control dysthymia. Research suggests that nutritional compounds may modulate depression-related biomarkers and may play a preventive or therapeutic role in the development and progression of depression.

Copper is an essential element in mammalian nutrition and acts as an electron donor or acceptor for multiple metalloenzymes (5). Its main biological functions include maintaining hematopoietic function, affecting energy metabolism, and influencing neurobehavioral and immune function. Previous studies suggest that increased levels of blood copper may be associated with depressive disorder, indicating a potential role for copper as a biomarker of depression (6–9).

A growing body of research has provided evidence that depression may be linked to folate deficiency (10, 11). Specifically, studies have shown that folate deficiency is associated with an increased risk of depression, more severe depressive symptoms, longer depressive episodes, and an increased risk of relapse of depressive symptoms. Some studies have also found that patients with depression had significant improvement after folic acid supplementation (12–14).

The inclusion of folic acid in American foods and the widespread use of folic acid supplements by many individuals have not eliminated the observed association between depressive symptoms and levels of folate and copper. This suggests that in countries and regions where mandatory folic acid fortification is not practiced, or where copper levels in the diet are naturally high, the correlation may be even more pronounced. Understanding the role of nutrients in the development of depressive symptoms, as well as the intricate interactions among various nutrients, remains a largely unexplored area. Previous cytological studies have observed that folate-deficient rat liver cells are more susceptible to copper toxicity (15). This suggests that there may be a synergistic effect between high blood copper levels and low folate levels. However, this hypothesis is not supported by population-based data. To address this knowledge gap, our study aimed to investigate whether serum folate levels can independently and jointly with serum copper levels affect the risk of depression using data from the National Health and Nutrition Examination Survey (NHANES).

## Methods

### Study design and population characteristics

NHANES is a national stratified sampling survey conducted by the National Center for Health Statistics (NCHS) of the Centers for Disease Control and Prevention (CDC). Its main purpose is to understand the health and nutritional status of the American population. The survey data is released every 2 years, and detailed survey operation manuals, consent documents, and brochures for each period can be found on the NHANES website. The NHANES was approved by the NCHS Institutional Review Board, and all participants signed an informed consent form.

**Figure 1:**
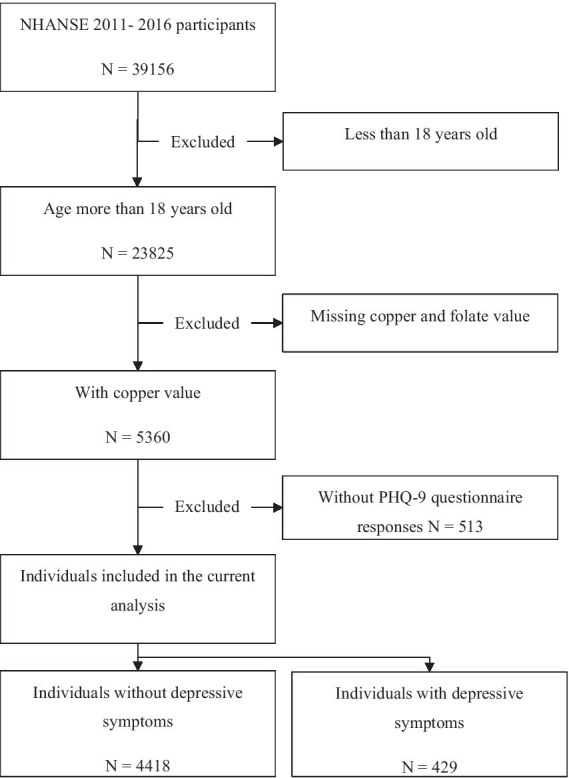
Flowchart.

The NHANES 2011–2016 database includes a total of 39,156 participants. We limited our analysis to individuals aged 18 years or older. Of the 23,825 participants, 18,978 were excluded due to missing information on copper, folate levels and PHQ-9 questionnaire responses. After excluding individuals with missing data sets, 4,847 participants were included (Figure 1).

### Data collection and processing

Specimen donors were advised to fast before specimen collection, but fasting was not mandatory. Details on fasting before blood draw were collected via questionnaire prior to the blood draw. Serum copper levels in NHANES participants were measured using inductively coupled plasma-dynamic reaction cell mass spectrometry (ICP-DRC-MS), with gallium as the internal standard. The lower limit of detection (LLOD) for serum copper was 2.5 μg/dL. None of the samples measured were below the LLOD in the final data (Cu_Min_: 24.7 μg/dL, Cu_Max_: 306.6 μg/dL). Serum total folate levels were also using LC–MS/MS by the CDC laboratory.

The data were returned to NHANES, and any incomplete data or improbable values were sent to the performing laboratory for clarification. Bench quality control (QC) methods were used to evaluate the accuracy and precision of the analytical process and to determine if the analytical system was “in control.” QCs were performed at the beginning and end of each analytical run. The analyte concentrations in the “low QC” were in the low-normal concentration range, while those in the “high QC” were in the high-normal concentration range. The QC summary indicated that the high QC coefficient of variation (CV) range for copper was 2.9–4.4, while the low QC CV range was 2.7–4.2. The high QC CV range for zinc was 5.4–6.1, while the low QC CV range was 4.8–5.7. Quality control procedures were consistent across cycles, and long-term quality control CVs were <3%. For more details on quality control, see the NHANSE website (16–20).

### Ascertainment of depression

Depression was assessed using a nine-item Patient Health Questionnaire (PHQ-9), which is a reliable and valid diagnostic tool for detecting depression in both clinical and research settings (21). Diagnostic and Statistic Manual of Mental Disorders (DSM-IV)-based symptom criteria for the nine-item instrument included “not at all,” “several days,” “more than half the days,” and “nearly every day,” with points ranging from 0 to 3 assigned to each response. A total score was calculated by summing the points for each item, with scores ranging from 0 to 27. A PHQ-9 score of ≥10 was used as the cut-off point to identify depression, with a sensitivity of 88% and a specificity of 88% for diagnosing major depression (22).

### Assessments of covariates

Demographic characteristics such as age, gender, estimated glomerular filtration rate (eGFR), history of diabetes, ethnicity (Mexican American, other Hispanic, Non-Hispanic White, Non-Hispanic Black, and other race), and education level (less than 9th grade, 9th–11th grade, high school graduate/GED or equivalent, some college or AA degree, and college graduate or above) were obtained from in-person household interviews. Education level was divided into <high school, high school, and >high school. History of diabetes was defined as a self-reported physician diagnosis of diabetes. Serum creatinine was measured using a kinetic rate Jaffe method in the mobile examination center. The metabolism of trace metal elements such as copper and zinc in the human body requires the kidneys, and glomerular filtration rate (GFR) is a widely accepted indicator of renal excretion function. Therefore, incorporating eGFR into the model correction helps to understand the independent effects of trace metal elements and depressive symptoms in populations with different levels of renal function. The eGFR was calculated using the Chronic Kidney Disease Epidemiology Collaboration (CKD-EPI) equation (23). Body mass index (BMI) was calculated as Weight (Kg)/Height (m) (2). History of cigarette smoking was defined as people used tobacco/nicotine last 5 day (Smoking questionnaire, SMQ680). Alcohol using was defined as people had at least 12 alcohol drinks/1 year (Alcohol use questionnaire, ALQ 101). Physical activity status was defined as the variable PAD675 from Physical Activity questionnaire (Minutes moderate recreational activities). Sleep disorder was defined as people ever told doctor had trouble sleeping (Sleep disorder questionnaire SLQ050). Previous studies have shown that there are common physicochemical pathways between copper and zinc, and their ratio is associated with endpoints such as sleep disturbances, cancer risk, and organ aging. Based on this relationship, we included zinc in the data analysis process to observe whether it exerts an influence on the results of this study.

### Statistical analysis

Data are presented as mean ± standard deviation (SD) for continuous variables and as proportions (%) for categorical variables. All continuous variables have passed the test for homogeneity of variance and conform to a normal distribution. Differences in characteristics between cases and controls were compared using *t*-tests for continuous variables and chi-square tests for categorical variables. Given the potential non-linear relationship between nutrients and outcomes and unknown cut-off values, odds ratios (ORs) and 95% confidence intervals (95% CIs) for depression were estimated by modeling nutrients as continuous variables and as quartiles using logistic regression. To further improve statistical power, if there was a significant relationship between nutrients and outcomes, quartiles were further pooled as binary variables. Multivariate adjustments were made for age, gender, body mass index, ethnicity, education levels, diabetes mellitus, self-reported sleep disorder, moderate recreational activities, history of alcohol consumption and cigarette smoking.

In addition, the interaction based on the additive model was evaluated using three indices: the relative excess risk of interaction (RERI), attributable proportion of interaction (API), and synergy index (SI). If 0 was contained within the 95% CI of RERI and API or if 1 was included in the 95% CI of SI, there was no interaction. Data were analyzed using R software (version 4.0.0; The R Foundation). A two-tailed *p*-value <0.05 was considered statistically significant in all analyses.

## Results

### Characteristics of enrolled participants

Table 1 shows the characteristics of the analyzed sample by depression status. Of the 4,847 participants, the prevalence of depression (PHQ-9 scale ≥10) was 8.9%. There were significant differences between participants with and without depression in terms of age, race, gender, eGFR, BMI, education level, cigarette smoking, self-reported sleep disorder, moderate recreational activities and history of diabetes. Compared to participants without depression, those with depression were more likely to be female, older, non-Spanish white race, have a history of diabetes, have lower eGFR, rate of alcohol drinking, levels of education and higher BMI, rate of sleep disorder, cigarette and moderate activities.

**Table 1:** Characteristics of the participants stratified by having depression symptoms or not.

	Have depression symptoms (PHQ-9 score ≥ 10)		*p*-values
	No	Yes	
*n*	4,418 (91.1)	429 (8.9)	
Age, years, mean (SD)	47.42 (18.6)	49.47 (17.2)	0.028
Male, %	2,253 (51.0)	174 (40.6)	<0.001
eGFR, mean (SD)	96.19 (24.5)	91.75 (27.2)	<0.001
BMI, mean (SD)	28.9 (6.9)	31.2 (8.8)	<0.001
Total folate, μmol/L	43.75 (28.4)	40.26 (26.6)	0.015
Serum zinc, μmol/L	12.48 (2.3)	12.40 (2.3)	0.494
Serum copper, μmol/L	18.63 (4.8)	19.62 (4.5)	<0.001
Self-report diabetes, yes, %	536 (12.1)	101 (23.5)	<0.001
Cigarette smoking, yes, %	747 (42.2)	161 (62.4)	<0.001
Alcohol drinking, yes, %	4,415 (99.9)	426 (99.8)	0.419
Self-report sleep disorder, yes, %	992 (22.5)	266 (62.0)	<0.001
Moderate recreational activities, minutes, mean (SD)	69.2 (236.0)	144.1 (912.4)	0.013
Education (%)			<0.001
High	2,415 (57.8)	167 (40.6)	
Low	870 (20.8)	145 (35.3)	
Mid	896 (21.4)	99 (24.1)	
Ethnicity, %			0.010
Mexican American	633 (14.3)	58 (13.5)	
Other Hispanic	462 (10.5)	62 (14.5)	
Non-Hispanic White	1715 (38.8)	169 (39.4)	
Non-Hispanic Black	940 (21.3)	97 (22.6)	
Other Race	668 (15.1)	43 (10.0)	

eGFR, estimated glomerular filtration rate; BMI, Body mass index.

### Relationship of serum copper and folate with the risk of depression

**Figure 2:**
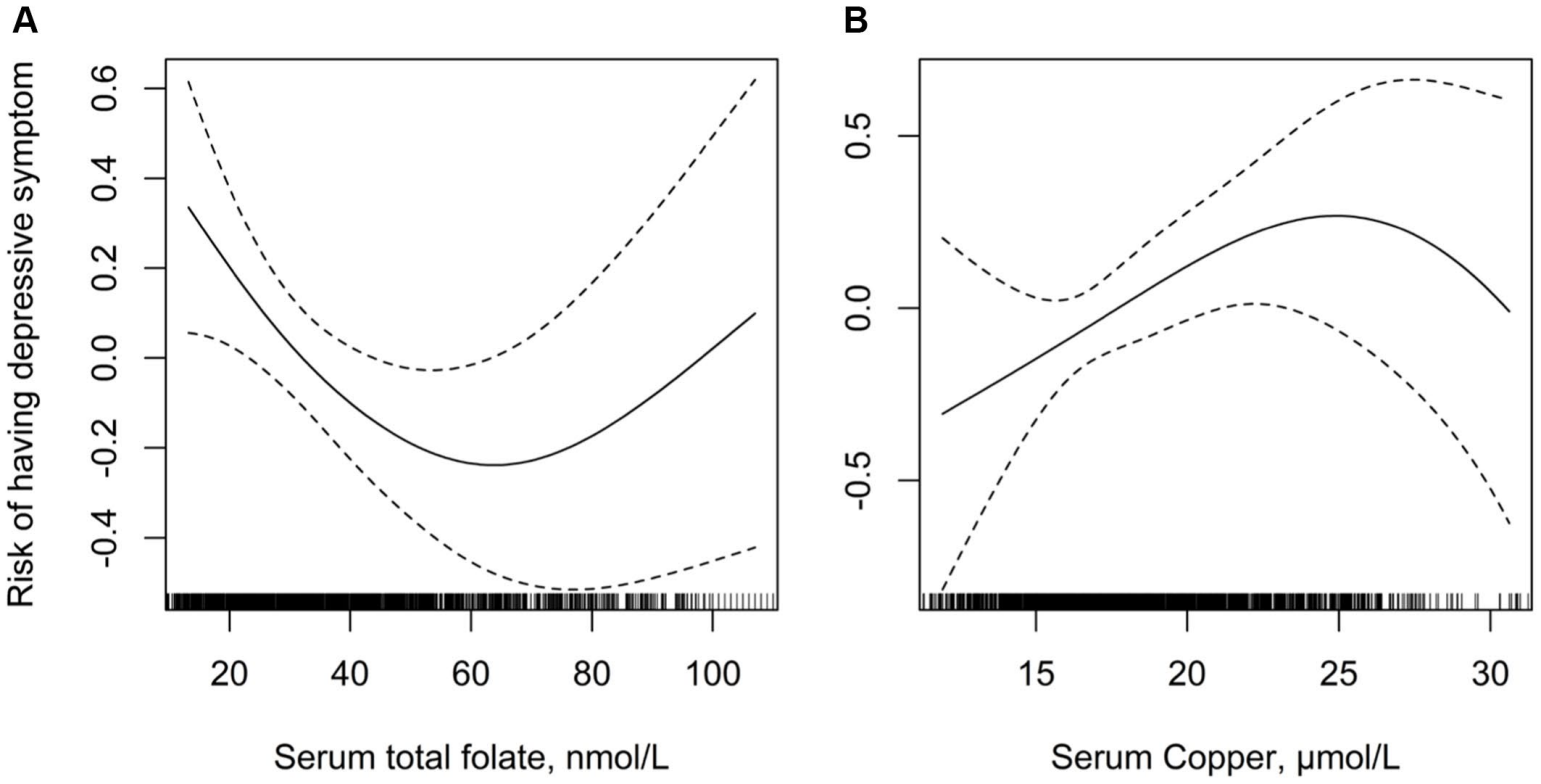
Multivariate adjusted association between folate and serum copper concentrations with depression. **(A)** Serum folate and risk of depression; **(B)** serum copper and risk of depression. Adjusted for age, gender, body mass index, ethnicity, education levels, diabetes mellitus, self-reported sleep disorder, moderate recreational activities, history of alcohol consumption and cigarette smoking.

Overall, there was a significant negative relationship between total folate levels and the risk of depression (Figure 2A). In contrast, there was a significant inverse relationship between serum copper levels and the risk of depression (Figure 2B). Results of the regression analyses are presented in Table 2. Multivariate-adjusted analyses indicated a significant negative correlation between serum folate levels and the risk of depression (adjusted OR: 0.98; 95% CI: 0.97–0.99). When serum folate concentration was analyzed in quartiles, compared to participants in the fourth quartile (>53.7 μmol/L), the adjusted OR for participants in the first quartile (<25.7 μmol/L), second quartile (25.7–36.9 μmol/L), and third quartile (36.9–53.7 μmol/L) was 2.06 (95% CI: 1.34–2.76), 1.49 (95% CI: 1.33–2.12), and 1.31 (95% CI: 0.96–1.78), respectively. Lower folate levels (quartiles 1–3, <53.7 μmol/L) were associated with a 44% increased risk of depression (OR: 1.44; 95% CI: 1.21–1.78) compared to higher levels (quartile 4: ≥53.7 μmol/L).

**Table 2:** Logistic regression of the association between nutrition elements and having depression symptoms.

Main effect	Crude		Adjusted*	
	OR (95% CI)	*P*-value	OR (95% CI)	*P*-value
Folate continuous	0.98 (0.97, 0.99)	0.011	0.98(0.97, 0.99)	<0.001
Folate quartiles				
Q1 (<25.7)	1.70 (1.23, 2.44)	<0.001	2.06(1.34, 2.76)	<0.001
Q2 (25.7–36.9)	1.40 (0.94, 1.69)	0.088	1.49 (1.33, 2.12)	0.018
Q3 (36.9–53.7)	1.14 (0.82, 1.53)	0.334	1.31 (0.96, 1.78)	0.066
Q4 (≥53.7)	Ref (1.0)		Ref (1.0)	
Folate category				
Q1–3 (<53.7)	1.42 (1.13, 1.79)	0.006	1.44 (1.21, 2.10)	0.003
Q4 (≥53.7)	Ref (1.0)		Ref (1.0)	
Copper continuous	1.05 (1.01, 1.07)	<0.001	1.04 (0.99, 1.04)	0.022
Copper quartiles				
Q1 (<15.5)	Ref (1.0)		Ref (1.0)	
Q2 (15.5–18.0)	1.12 (0.88, 1.47)	0.433	1.08 (0.80, 1.57)	0.884
Q3 (18.0–21.0)	1.68 (1.33, 2.26)	<0.001	1.48 (1.11, 2.13)	0.032
Q4 (≥21.0)	2.04 (1.60, 2.77)	<0.001	1.71 (1.12, 2.34)	0.004
Copper category				
Q1 (<15.5)	Ref (1.0)		Ref (1.0)	
Q2–4 (≥15.5)	1.77 (1.39, 2.48)	<0.001	1.54 (1.18, 2.11)	0.005

*Multivariate logistic regression analysis adjusting for age, gender, body mass index, ethnicity, education levels, diabetes mellitus, self-reported sleep disorder, moderate recreational activities, history of alcohol consumption and cigarette smoking.

After adjusting for potential confounding factors, the risk of depression significantly increased with serum copper levels (OR: 1.04; 95% CI: 0.99–1.04). When serum copper levels were assessed in quartiles, a higher risk of depression was found in subjects in quartile 2 (15.5–18.0 μmol/L; OR: 1.08; 95% CI: 0.80–1.57), quartile 3 (18.0–21.0 μmol/L; OR: 1.48; 95% CI: 1.11–2.13), and quartile 4 (≥21.0 μmol/L; OR: 1.71; 95% CI: 1.12–2.34) compared to those in quartile 1 (<15.5 μmol/L). High copper levels (quartiles 2–4) were associated with a 54% increased risk of depression (OR: 1.54; 95% CI: 1.18–2.11) compared to lower levels (quartiles 1).

Additive interaction terms for copper and folate levels were constructed, including low copper and low folate, low copper and high folate, high copper and low folate, and high copper and high folate. Each interaction term is shown in Table 3. In univariate analysis, participants with high copper and low folate levels had a significantly elevated risk of depression compared to those with low copper and high folate levels (OR: 2.11; 95% CI: 1.43–3.14), and this risk persisted after adjusting for all confounders (OR = 2.01; 95% CI: 1.37–3.15). Compared to the control group (low copper and high folate), the risk of depression showed a general upward trend in participants with both high levels of copper and folate (OR: 1.26; 95% CI: 0.66–1.98), but the result was not statistically significant. Participants with both low levels of copper and folate also showed a similar upward trend (OR: 1.39; 95% CI: 0.89–2.15).

**Table 3:** Logistic regression analysis of the interactive items between folate and copper levels.

		Crude		Adjusted	
		OR (95% CI)	*P*-value	OR (95% CI)	*P*-value
Cu_High	Fo_Low	2.11 (1.43, 3.14)	<0.001	2.01 (1.37, 3.15)	<0.001
Cu_High	Fo_High	1.31 (0.80, 2.03)	0.177	1.26 (0.66, 1.98)	0.312
Cu_Low	Fo_Low	1.10 (0.77, 1.69)	0.380	1.39 (0.89, 2.15)	0.087
Cu_Low	Fa_High	Ref (1.0)		Ref (1.0)	
RERI (95% CI)		0.58 (0.07, 1.18)		0.43 (0.13, 1.02)	
API (95% CI)		0.29 (0.04, 0.55)		0.19 (0.01, 0.54)	
SI (95% CI)		2.18 (1.47, 8.36)		1.65 (1.49, 4.76)	

*Multivariate logistic regression analysis adjusting for age, gender, body mass index, ethnicity, education levels, diabetes mellitus, self-reported sleep disorder, moderate recreational activities, history of alcohol consumption and cigarette smoking. OR, odds ratio; 95% CI, 95% confidence interval; RERI, relative excess risk of interaction; API, attributable proportion of interaction; SI, synergy index. Cu, copper; Fo, total folate.

The logistic regression analysis indicated that the interactive indices in crude model and adjusted model were as follows: RERI_crude_ (0.58; 95% CI: 0.07–1.18), RERI_adjusted_ (0.43; 95% CI: 0.13–1.02); API_crude_ (0.29; 95% CI: 0.04–0.55), API_adjusted_ (0.19; 95% CI: 0.01–0.54); SI_crude_ (2.18; 95% CI: 1.47–8.36), SI_adjusted_ (1.65; 95% CI: 1.49–4.76). The 95% CIs of RERI and API suggested that there may be a synergistic interaction between high copper and low folate levels on the occurrence of depression. In addition, after adjusting for all confounders, the API was 0.19, indicating that the proportion of depression cases that may be caused by the interaction between copper and folate levels was 19.0% among all participants with depression.

## Discussion

Our study examined the main and interaction effects of serum copper and folate levels on the risk of depressive symptoms using data from the NHANES database. This study confirmed that high serum copper levels or low serum folate levels were independent risk factors for depressive symptoms and further found that serum copper and folate levels may have a synergistic interaction in the occurrence of depression. The proportion of depression cases that may be caused by the interaction between high serum copper and low folate levels was 21% among all patients with depressive symptoms.

### Main effect of high blood copper and low folate are associated with having depression symptom

There is growing evidence that depression may be linked to folate deficiency. Specific studies have shown that folate deficiency is associated with an increased risk of developing depression, more severe depressive symptoms, longer depressive episodes, and relapse of depressive symptoms. A meta-analysis found that individuals with depression had significantly lower levels of folate than those without depression (10). It has been suggested that people who are deficient in folate may also have relatively low levels of neurotransmitters such as dopamine, norepinephrine, and serotonin, which can lead to depressive symptoms. There is also some evidence that the biological mechanism by which folate deficiency is associated with depression may be related to homocysteine levels (24).

Copper has been recognized as an essential nutrient since the 1920s and is known to be a part of or a cofactor for about 30 enzymes and proteins (25, 26). Some of the copper-binding enzymes and proteins and their known functions have been identified. High serum copper levels are also an independent risk factor for depression. A recent meta-analysis showed that patients with depression had higher blood copper levels than controls without depression (5). The authors summarized possible mechanisms for the association between copper and depression, such as the monoamine hypothesis (DA, NE, 5-HT), receptor hypothesis, neuroendocrine system, and immune system. Despite increased understanding of the role copper plays in depression physiology and its potential contribution to several chronic diseases, the true pathogenesis has not been clearly elucidated.

### Interaction effect of high blood copper and folate deficiency on depression

Our findings suggest that high blood copper levels and folate deficiency have a synergistic effect, increasing the risk of depression. However, in the high folate group, the damaging effect of high blood copper levels was not significant. We speculate that the mechanism may be related to the following two aspects.

First, copper-induced impairment of mitochondrial respiratory function may be a key step in the cuproptosis process that may be blocked by folate. A recent study showed that copper-mediated cell death differs from known death mechanisms and is dependent on mitochondrial respiration (27). The study further clarified that copper-dependent death occurs through direct binding of copper to lipoylated components of the tricarboxylic acid (TCA) cycle. This results in lipoylated protein aggregation and subsequent loss of iron–sulfur cluster proteins, leading to proteotoxic stress and ultimately cell death. This mechanism is distinct from previously proposed theories such as apoptosis, ferroptosis, pyroptosis, and necroptosis and has been described as cuproptosis (5, 6, 28). It is known that elevated homocysteine levels can also adversely affect mitochondrial respiratory function (29). When incubated with aorta endothelial cells, homocysteine produced oxidative cell injury and lipid peroxidation as a result of mitochondrial toxicity (decreased respiration and RNA levels) induced by synergism between homocysteine and H_2_O_2_ generated by autoxidation. Supplementation with folic acid and vitamin B12 can promote the regeneration of methionine from homocysteine, suggesting that folate has the effect of maintaining the stability of mitochondrial respiratory function (27).

From this perspective, the increase in homocysteine levels caused by folate deficiency and high blood copper levels may have a compounded harmful effect on brain cells, potentially leading to a significant increase in the risk of depression. In the high folate group, it is understandable that mitochondrial respiratory function is protected by folate against the adverse effects of homocysteine, and the risk of depression may decrease accordingly (30).

Secondly, folic acid helps protect the blood–brain barrier and reduces the direct damage of copper to brain cells (14, 31). Copper is transported from the blood circulation into the brain via the blood–brain barrier (BBB) and blood-CSF barrier (BCB) (32). The BBB acts as the primary transport route for copper into the brain parenchyma, while the BCB mainly maintains copper homeostasis in the brain by exporting excess copper from the CSF to the blood. Copper concentration plays a crucial role in oxidative stress processes and influences the catalytic and structural properties of some antioxidant enzymes, which may be one of the main causes of depression development. A study demonstrated that folic acid protects BBB permeability, decreases neuroinflammation by reducing MPO activity, and decreases oxidative damage to lipids in brain structures acutely after sepsis induction (33). In the long term, its improved memory impairment and increased survival. Therefore, in the low folic acid group, copper ions were more likely to penetrate the blood–brain barrier and cause oxidative damage to brain cells. The high folic acid group may benefit from folic acid’s stable maintenance of the blood–brain barrier, reducing copper’s toxic effect on brain cells and thereby reducing the risk of depression.

### Strengths and limitations

Our study observed that high blood copper and low folate levels may interact to increase the risk of depression. However, the increased risk of depression associated with blood copper was not significant in people with high folate levels. The possible mechanism of the interaction between high blood copper and folic acid formation was also discussed. This study had several strengths, including its large and nationally representative sample, the use of a central laboratory and standardized assay, and the use of major associated factors in the statistical analysis. However, potential limitations of the current analysis should also be considered when interpreting the study results. Firstly, this study was a cross-sectional study that cannot confirm the causal relationship between the interaction of serum copper and folate on depression occurrence. Stronger evidence relies on prospective cohort studies. Secondly, a statistically significant interaction does not imply the existence of a biological interaction. We need more basic research to elucidate the mechanisms of possible interactions between folate and copper elements in the human body. Thirdly, the depression outcome in NHANES was based on self-reported data from participants. Individuals with high PHQ-9 scores only indicated having depressive symptoms and did not represent a diagnosis of depression. However, previous studies have shown that the PHQ-9 questionnaire is a clinically validated tool for assessing depression, and it has reliable specificity and sensitivity in screening for depressive patients (34). Fourthly, 9.3% (429/4847) of the respondents in this study exhibited depressive symptoms, resulting in a certain degree of imbalance between groups in the data. As a result, we employed a multi-factor correction method to minimize the impact of this factor.

In summary, depressive symptoms vary widely in prevalence and severity across individuals, ranging from mild manifestations such as sleep disturbances and decreased concentration to severe cases that can lead to suicidal. Current treatment approaches, including antidepressant medications and psychological interventions can be accompanied by significant side effects. The emerging research exploring the link between nutrients and depressive symptoms offers a promising alternative or adjunctive approach to traditional treatments. By identifying specific nutrients that may play a role in modulating mood and cognitive function, it may be possible to develop safer and more cost-effective strategies for reducing the residual risk of depression. If further research confirms that balancing certain nutrient levels can help alleviate depressive symptoms, this could lead to the development of personalized nutrition plans tailored to individual needs. Such an approach could not only improve mental health outcomes but also reduce reliance on pharmacological treatments and their associated side effects.

## Conclusion

There may be a synergistic interaction between high copper levels and low folate levels on prevalence of depressive symptoms. Further population-based interventional studies are needed to confirm whether folic acid supplementation is effective in preventing depression in individuals with high blood copper levels.

## Data Availability

Publicly available datasets were analyzed in this study. This data can be found: National Health and Nutrition Examination Survey 2013–2014 Data Documentation, Codebook, and Frequencies Copper, Selenium and Zinc — Serum (CUSEZN_H), https://wwwn.cdc.gov/Nchs/Nhanes/2013-2014/CUSEZN_H.htm.
