# Supplementary material for: Independent and combined effect of serum copper and folate on depression: cross-sectional data from the NHANES 2011–2016
Source: Front Nutr. 2024 Aug 9;11:1389480. doi: 10.3389/fnut.2024.1389480 (PMC11341402; doi:10.3389/fnut.2024.1389480)
Supplement: Supplementary file 1 [file Table_1.DOCX]

**Supplemental table 1. Weighted data stratified by having depression symptomes or not.**

|  | Have depression symptomes  (PHQ-9 score≥10) | | *P-values* |
| --- | --- | --- | --- |
|  | No | Yes |  |
| n | 60669753.12 | 5024853.97 |  |
| Age, years, mean (SD) | 46.65 (17.57) | 47.11 (16.46) | 0.686 |
| Male, % | 30606336.4 (50.4) | 2064818.2 (41.1) | <0.001 |
| eGFR, mean (SD) | 46.65 (17.57) | 47.11 (16.46) | 0.686 |
| BMI, mean (SD) | 28.89 (6.77) | 30.80 (8.25) | <0.001 |
| Total folate, μmol/L | 44.93 (27.49) | 41.85 (26.6) | <0.001 |
| Serum zinc, μmol/L | 12.57 (2.34) | 12.49 (2.14) | 0.494 |
| Serum copper, μmol/L | 18.41 (4.77) | 19.41 (4.22) | <0.001 |
| Self-report diabetes, yes, % | 5991329.3 ( 9.9) | 942284.1 (18.8) | <0.001 |
| Cigarette smoking, yes, % | 9720209.3 ( 39.0) | 2325412.8 (69.4) | <0.001 |
| Alcohol drinking, yes, % | 60618998.6 (100.0) | 4986460.7 (99.4) | <0.001 |
| Self-report sleep disorder, yes, % | 15658447.1 ( 25.8) | 3193991.9 (63.6) | <0.001 |
| Moderate recreational activities, minutes, mean (SD) | 65.27 (131.43) | 135.45 (895.20) | 0.391 |
| Education (%) |  |  | <0.001 |
| High | 38935584.8 (66.2) | 2347736.9 (48.3) |  |
| Low | 7789478.3 (13.2) | 1221876.9 (25.1) |  |
| Mid | 12065430.8 (20.5) | 1291063.7 (26.6) |  |
| Ethnicity, % |  |  | 0.010 |
| Mexican American | 5332242.2 ( 8.8) | 393452.9 ( 7.8) |  |
| Other Hispanic | 3493436.4 ( 5.8) | 415240.6 ( 8.3) |  |
| Non-Hispanic White | 40914244.9 (67.4) | 3174040.6 (63.2) |  |
| Non-Hispanic Black | 6228461.4 (10.3) | 671956.1 (13.4) |  |
| Other Race | 4701368.2 ( 7.7) | 370163.7 ( 7.4) |  |

**Abbreviations**: eGFR, estimated glomerular filtration rate; BMI, Body mass index.
